# Acute Coronary Syndrome as an Unusual Initial Presentation of T-Prolymphocytic Leukemia: A Case Report and Review of the Literature

**DOI:** 10.1155/2024/4303614

**Published:** 2024-08-27

**Authors:** Christian R. Klein, Felix Jansen, Peter Brossart, Marco Herling, Georg Feldmann

**Affiliations:** ^1^ Department of Oncology, Hematology, Immune-Oncology and Rheumatology University Hospital Bonn, Bonn, Germany; ^2^ Center for Integrated Oncology Aachen Bonn Cologne Duesseldorf (CIO ABCD), Bonn, Germany; ^3^ Department of Internal Medicine II University Hospital Bonn University of Bonn, Venusberg-Campus 1, Bonn 53127, Germany; ^4^ Department of Hematology, Cellular Therapy, and Hemostaseology University of Leipzig, Leipzig, Germany

## Abstract

T-prolymphocytic leukaemia (T-PLL) is the most common mature T-cell leukaemia in Central Europe and is often manifested by rapidly increasing lymphocytosis, marked bone marrow infiltration and splenomegaly. In 10–15% of cases, the diagnosis is made by incidental findings in otherwise asymptomatic patients. Here we report a case of T-PLL that initially became symptomatic due to the presence of acute coronary syndrome (ACS). Initially, emergency coronary angiography with consecutive emergency 5-coronary artery bypass grafting (CABG) was performed. Leukocytosis was found perioperatively and T-PLL (with TCL1 rearrangement) was subsequently diagnosed. Despite known potential cardiotoxicity, the patient was treated with the anti-CD52 antibody alemtuzumab with a gradual dose increase from 3 mg to 30 mg per day. Systemic alemtuzumab therapy resulted in the complete remission of T-PLL in the bone marrow without any impairment to cardiac function. The patient was then eligible to undergo a consolidating allogeneic stem cell transplant (alloSCT). The reported case shows that T-PLL can also become initially symptomatic through an acute coronary syndrome on the basis of complex coronary heart disease. Targeted antileukaemic therapy with the monoclonal antibody alemzutumab can lead to effective systemic cytoreduction without cardiac dysfunction even in patients with severe cardiac disease, although cases of cardiotoxicity have been reported.

## 1. Background

T-prolymphocytic leukaemia (T-PLL) is the most common mature T-cell leukaemia in Central Europe with an incidence of ≈2.0/million. It often presents with rapidly increasing lymphocytosis, marked bone marrow infiltration, and splenomegaly [1]. In approximately 10–15% of cases, the diagnosis is an incidental finding in otherwise asymptomatic patients. Current pathogenetic disease concepts include constitutive activation of T-cell leukaemia 1 (TCL1) oncogenes due to defective rearrangements with T-cell receptor (TCR) genes in combination with deleterious ATM aberrations [2]. The neoplastic cells present as mature, postthymically differentiated lymphocytes and usually carry a memory phenotype [3]. Given the very low incidence of T-PLL and the limited data sets of documented cases, no exogenous risk factors have yet been identified. T-PLL is also not a typical secondary neoplasia after cytostatic or immunomodulatory therapy. Medium age at first diagnosis is approximately 65 years, increased incidence rates have been described in younger patients with autosomal recessive ataxia telangiectasia (Louis–Bar syndrome), which represent compound heterozygous individuals for biallelic inactivating mutations of the ATM tumor suppressor gene [4, 5].

Clinically, T-PLL often presents with nonspecific symptoms such as fatigue, infections, bleedings, and B-symptoms in the context of a marked lymphocytosis, which in 75% of patients is found to exceed 100,000/*μ*l and often increases at an exponential rate [6]. Bone marrow infiltration or splenomegaly is observed in approximately 80% of cases. Frequent further findings include small-node generalized lymphadenopathies (45–50%), thrombocytopenias (<100,000/*μ*l in 45–50%), hepatomegaly (35–40%), anemias (<10 g/dL in 25%), skin infiltrations (25%), and malignant effusions (10–15%; pleura, pericardium, ascites) [7]. Established major diagnostic criteria are blood monoclonal lymphocytosis (>5 × 10^3^/*μ*l) of mature T-cell immunophenotype and evidence of a chromosomal aberration involving the loci 14q32.1 (TCL1A) or Xq28 (MTCP1) or evidence of T-cell-specific expression of TCL1A or MTCP1 protein by flow cytometry or immunohistochemistry. Secondary criteria include rapidly increasing blood lymphocyte counts with doubling times <6 months, additional evidence of chromosomal aberrations with gains on 8q, del11q22, or complex aberrant karyotype, the presence of effusions or (hepato)splenomegaly, or prolymphocytic morphology on blood smear [6, 8].

Due to its high efficacy, intravenous therapy with the anti-CD52 antibody alemtuzumab is a currently widely accepted first-line therapeutic standard [1]. The only curative treatment option to date is subsequent allogeneic stem cell transplantation (alloSCT), in which 15–30% of patients achieve remission that lasts longer than 4-5 years. Due to the high one-year mortality after alloSCT and with only 30–50% of patients being eligible for it, median overall survival across all patients is <2 years, and the 5-year survival rate is <5% [9].

Here, we report a case of T-PLL who initially became symptomatic with acute coronary syndrome (ACS) due to high-grade coronary three-vessel disease. Following coronary artery bypass grafting (CABG), in which five grafts were employed to treat triple vessel disease, antileukaemic therapy with alemtuzumab led to effective systemic cytoreduction without cardiac dysfunction although cases of cardiotoxicity have been reported. The case shows ACS as an unusual first symptom of T-PLL and demonstrates that alemtuzumab therapy (in an adapted, steady dose increase) can also be an effective induction therapy before alloSCT in patients with severe cardiac disease.

## 2. Case Presentation

A 66-year-old male patient presented at the emergency department of a peripheral hospital in February 2021 with suspected subacute myocardial infarction and angina pectoris symptoms that had persisted for one week. The patient had no known cardiovascular risk factors. Due to ST-segment depressions in II, III, aVF, and V2-V6 of the 12-channel ECG, immediate coronary angiography was performed. It revealed severe coronary triple-artery disease and the patient were transferred to cardiac surgery for emergency myocardial revascularization. A 5-fold CABG (LIMA ad LAD, V ad PDA, V ad M1 (first marginal branch of the circumflex ramus), V and D1, V as T-graft ad M1 and LAD; description in the list of abbreviations) was performed under hypothermic extracorporeal circulation. Intraoperatively, a markedly enlarged heart with hypokinesis of the posterior wall was seen with preserved pump function without arteriosclerosis of the ascending aorta. Given the calcification of the left anterior descending artery (LAD) throughout its length, along with the limited explorability of only approximately 1.5 cm proximally and in the distal third, a LIMA-LAD bypass and a venous T-graft at M1 and LAD were deemed the most appropriate options. Flow measurement of the grafts showed acceptable flows to the target vessels (LIMA-LAD: 80 ml/min, V-LAD: 60 ml/min, M1: 60 ml/min, D1 26 ml/min, PDA: 80 ml/min) so that surgery could be finalized without transfusion of blood products. Apart from a paranasal basal cell carcinoma, excised in 2020, no other previous diseases were present. Clinically, there were no signs of infection, no fever, no pain, and no signs of bleeding. No B-symptoms were detected, although slight night sweats had occurred in the days following the operation. The patient had not taken any long-term medication prior to his hospitalisation. There were no indications of malignant diseases in the family, the patient's mother had suffered a myocardial infarction. The patient was a nonsmoker and only occasionally consumed a small amount of alcohol. He had worked as an engineer in a chemical company before his retirement and had no occupational exposure to potentially toxic substances. The emergency situation described occurred acutely without a previous cardiological medical history and without previous cardiological events.

The initial blood count in the emergency department showed haemoglobin 14.1 g/dl, 30.60 G/l leucocytes, 151 G/l platelets, 14.08 G/l lymphocytes, 0.92 G/l monocytes, 9.79 G/l segmental nucleated neutrophils, 0.31 G/l basophils, and 0.31 G/l eosinophils.

On the first postoperative day, perioperative antibiotic treatment with cefuroxime (parenteral 1500 mg/100 ml i.v. over 24 h) resulted in a leucocytosis of 41.7 G/l and a subfebrile temperature of 36.9°C. There was no serological evidence of an acute viral infection (hepatitis B, C, HIV) or reactivation (CMV, EBV, and HSV).

**Figure 1:**
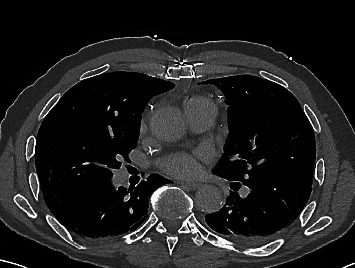
Exemplary transverse section of the staging CT image at initial diagnosis: the number of thoracic lymph nodes is clearly increased.

Contrast-enhanced computed tomography (CT, neck/thorax/abdomen, Figure 1) showed increased, partially enlarged lymph nodes, bilaterally cervical, axillary, retroperitoneal/paraaortic, and inguinal, as well as a slightly plump, discretely enlarged spleen (pole-to-pole 11.5 cm) with homogeneous parenchyma. There was no evidence of pericardial effusion on imaging.

Due to the increasing leukocytosis, treatment with prednisolone and vincristine was initiated before the diagnosis was completed under the suspected diagnosis of acute lymphoblastic T-cell leukaemia (T-ALL), which was well tolerated by the patient.

**Figure 2:**
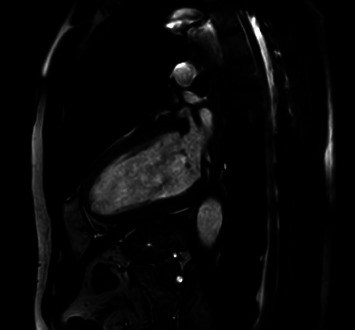
Cardiac MRI imaging at initial diagnosis after cardiovascular bypass surgery: infarct scar (over 75% transmural) on the left ventricular posterior wall and a normally dimensioned left ventricle with significantly reduced left ventricular function.

Cardiac involvement was suspected by cardiac magnetic resonance imaging (MRI), which revealed a postischaemic scar of the left ventricular posterior wall (more than 75% transmural) and a normally sized left ventricle with significantly reduced left ventricular function (LVEDV/BSA: 85 ml/m^2^, LVEF: 38%) without higher-grade valvular vitiation (Figure 2).

**Figure 3:**
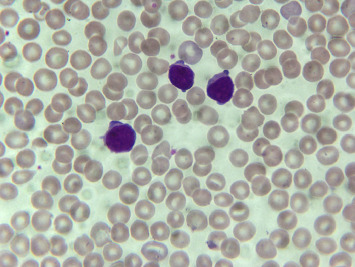
Cytomorphological findings in peripheral blood at initial diagnosis (magnification 100x): blood smear showing aberrant lymphoid cells with partially scored nuclei, including prominent nucleoli, and deep basophilic cytoplasm with multiple small protrusions (blebs) consistent with T-PLL.

**Figure 4:**
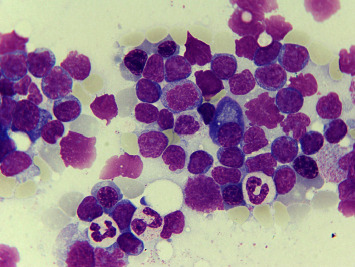
Findings in bone marrow at initial diagnosis (magnification 100x): prominent infiltration (approx. 70% of nucleated cells) by atypical small T-cells with prolymphocytic morphology. Lymphoid-appearing cells with mostly mature nuclear chromatin and slightly enlarged and shape-variable nuclei. In addition, repressed myelopoiesis with slightly increased eosinophilic granulocytes.

By flow cytometry and cytogenetics, the diagnosis of T-PLL could be established with a main criterion of mature T-cell immunophenotype and a secondary criterion of chromosomal aberration with an increase to 8q in 03/21. The cytomorphology characteristic of T-PLL is shown as an example in Figures 3 and 4. With stable leukocyte counts after prednisolone and vincristine, a temporary watch and wait approach was adopted until final diagnosis confirmation and cardiological rehabilitation. A cardiological follow-up examination in 05/21 revealed preserved left ventricular function on echocardiography with an ejection fraction (EF) of 57.1% without higher-grade vitiation, pericardial effusion, and clinically without cardiac symptoms.

In 06/21, the diagnosis of T-PLL (stage IV Ann Arbor) was finally confirmed by detection of TCL1a rearrangement in peripheral blood lymphocytes using FISH. CT-based staging showed a discrete increase in the size of the cervical, axillary, abdominal and inguinal lymph nodes as well as increasing splenomegaly.

**Figure 5:**
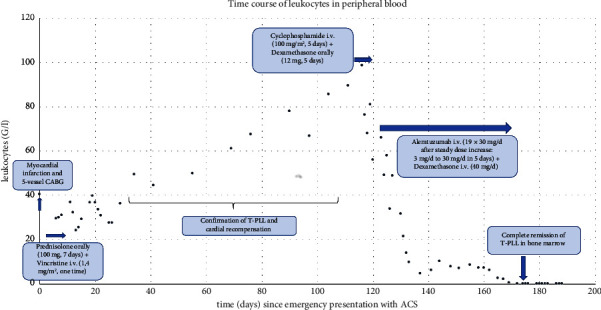
Kinetics of leukocytes in peripheral blood (G/l, measured by flow cytometry, reference range 3.6–10.5 G/l) over time (days) since emergency presentation with ACS.

After a continuous increase in the leucocyte count to 90 G/l at the end of 06/21 (Figure 5), the patient received cytoreductive therapy with cyclophosphamide/dexamethasone (200 mg/12 mg absolute/day) for five days. The reasoning underlying this preinduction phase was to minimise the potential cardiac risks described for alemtuzumab. This prephase of therapy was tolerated without complications. Alemtuzumab was then administered intravenously in steadily increasing doses (doses d1: 3 mg, d3: 10 mg, d5: 30 mg). After the first two doses, there was a progressive increase in serum creatinine (presumably in connection with tumour lysis), which rapidly subsided under supportive treatment. On day 5, administration of the 30 mg dose resulted in an anaphylactic reaction with chills, fever, drop in blood pressure, and severe nausea, which was effectively controlled by treatment with H1 and H2 blockers and glucocorticoids. A further dose of 30 mg was well tolerated with extended premedication with dexamethasone 40 mg, so that premedication with dexamethasone was continued for the next doses.

With continued good tolerability, the therapy was continued on an outpatient basis in 07/21. During treatment in 07/21 and 08/21, CMV reactivation occurred (specific nucleic acid amplificates: 3,640 IU/ml), which was treated with valganciclovir (900 mg bid) as an outpatient, as well as fungal pneumonia, which was treated as an inpatient with liposomal amphotericin B and piperacillin/tazobactam (consecutive switch to meropenem). A new bone marrow biopsy was performed in 08/21, which revealed a hypoplastic bone marrow with cytological complete remission of T-PLL. At the time of completion of this case report, the patient was in a good general condition and was able to continue the planned therapy with alemtuzumab until the consecutive consolidative alloSCT (MUD-PBSCT, conditioning: fludarabine 30 mg/m^2 KOF and TBI) in 11/21. A follow-up echocardiogram in 02/22 showed a largely preserved left ventricular function with an ejection fraction (EF) of 49% without higher-grade ventricular ejection, electrocardiography showed a normo-frequent sinus rhythm without specific pathologies.

## 3. Discussion

The case presented here is noteworthy due to ACS as the first symptom of T-PLL and alemtuzumab therapy in the presence of severe underlying cardiological disease.

### 3.1. ACS as Initial Symptom of T-PLL

ACS as first symptom of T-PLL is highly uncommon. To our knowledge, this is the first case report of ACS as an initial symptom of T-PLL. In other types of leukaemia, ACS has been described as the first clinical symptom in some case reports [9–20]. Some case reports have also reported T-PLL with cardiac involvement [21–24]. Case reports to date also describe any degree of cardiac symptoms up to and including cardiac arrest as the initial symptom of cardiac infiltration [18]. Furthermore, several case reports have documented the occurrence of pericardial and myocardial leukaemia infiltration in instances of ALL recurrence subsequent to alloSCT [25–27]. The main pathomechanisms underlying ACS in the more common acute and chronic leukaemias are myocardial ischaemia due to anaemia, rheological disturbances due to leukostasis associated with hyperleukocytosis and hypercoagulable states [28]. A systematic review of case reports with cardiac infiltration as the first symptom in acute lymphoblastic leukaemia can be found in [29]. In the case presented here, global clotting times were in the normal range, leukocytosis was only moderate (30,000/*μ*l), and the haemoglobin level was 14.1 mg/dl. The ACS developed on the basis of a complex three-vessel disease with moderate leukocytosis and increasing kinetics. The case presented here demonstrates that in the presence of coronary artery disease, ACS can occur in the manifestation of leukaemia even with only moderate leukocytosis. In this sense, a previous cardiological or angiological disease may lower the leukocyte threshold for a leukostasis syndrome. For this reason, it may make sense to initiate cytoreductive therapy early in the diagnosis of leukaemia in the presence of a positive cardiological history and, on the other hand, to include an underlying haematological disease in the differential diagnosis of ACS, even in the presence of only moderate leukocytosis, as in the case presented here.

The neutrophil to lymphocyte ratio (NLR) has proven to be a useful predictive marker for identifying high-risk ACS patients with a poor prognosis [30, 31]. It should be noted that patients with malignant haematological diseases as the underlying cause of acute cardiac symptoms may represent an exception to this rule, and that NLR may not be a suitable biomarker in such cases [32, 33].

### 3.2. Alemtuzumab in Patients with Severe Preexisting Cardiological Disease

Alemtuzumab is a humanised IgG1*κ* monoclonal antibody that binds specifically to the CD52 glycoprotein (CAMPATH 1 antigen), which is expressed on mature B and T lymphocytes. The antibody is used in the treatment of multiple sclerosis, chronic lymphocytic leukaemia (CLL), T-PLL, and for induction therapy in kidney transplantation [34]. Cardiotoxicity of alemtuzumab has been described in several case reports [35–38]. In the TPLL1 study [39], there was also one documented death due to myocardial infarction, which was presumably attributable to the cardiotoxicity of alemtuzumab. In the case presented here, the T-PLL initially presented as ACS in the setting of severe coronary artery disease. Despite a risk of further cardiac events, early cytoreduction with cyclophosphamide/dexamethasone prior to definitive alemtuzumab induction proved successful, as it was tolerated without complications and may have reduced cardiac risk. In addition to reducing the overall leukaemic burden and thus alemtuzumab-induced cytokine release, it is also known that cyclophosphamide positively modulates the local macrophage milieu towards alemtuzumab sensitisation [40]. The approach described here may therefore also serve as a guide for the treatment of similar future cases of T-PLL with severe preexisting cardiac disease.

## Data Availability

No data were used to support this study.

## Abbreviations

ACS: Acute coronary syndrome
alloSCT: Allogeneic stem cell transplantation
BSA: Body surface area
CABG: Coronary artery bypass grafting
CLL: Chronic lymphatic leukaemia
CT: Computed tomography
ECG: Electrocardiogram
EF: Ejection fraction
FISH: Fluorescence in situ hybridization
LAD: Left anterior descending artery
LIMA: Left internal mammary artery
LVEDV: Left ventricular end-diastolic volume
LVEF: Left ventricular ejection fraction
M1: First marginal branch of the circumflex ramus
MRI: Magnetic resonance imaging
NLR: Neutrophil to lymphocyte ratio
PDA: Posterior descending artery
PET-CT: Positron emission tomography-computed tomography
T-ALL: T-cell acute lymphoblastic leukaemia
TCL1: T-cell leukaemia 1
TCR: T-cell receptor
T-PLL: T-prolymphocytic leukaemia
TTE: Transthoracic echocardiogram.
